# Music Tempo: A Tool for Regulating Walking Cadence and Physical Activity Intensity in Overweight Adults?

**DOI:** 10.3390/ijerph18157855

**Published:** 2021-07-25

**Authors:** Maria Faulkner, Andrea McNeilly, Gareth Davison, David Rowe, Allan Hewitt, Alan Nevill, Ellie Duly, Tom Trinick, Marie Murphy

**Affiliations:** 1 Sports Lab North West, Letterkenny Institute of Technology, F92 FC93 Donegal, Ireland; 2 Sport and Exercise Sciences Research Institute, Ulster University, Newtownabbey BT37 0QB, UK; a.mcneilly@ulster.ac.uk (A.M.); gw.davison@ulster.ac.uk (G.D.); mh.murphy@ulster.ac.uk (M.M.); 3 School of Psychological Sciences and Health, University of Strathclyde, Glasgow G1 1XQ, UK; darowe999@gmail.com (D.R.); allan.hewitt@strath.ac.uk (A.H.); 4 Faculty of Education Health & Wellbeing, University of Wolverhampton, Walsall WS1 3BD, UK; A.M.nevill@wlv.ac.uk; 5 Clinical Biochemistry Department, Ulster Hospital, South Eastern Health Trust, Belfast BT16 1RH, UK; Ellie.Duly@setrust.hscni.net (E.D.); Tom.Trinick@setrust.hscni.net (T.T.)

**Keywords:** individualized physical activity, stride rate guidelines, beats per minute, physical activity guidelines, health

## Abstract

This study investigated if music tempo can prompt a desired walking cadence, and if music can provide a stimulus to regulate physical activity intensity in a longitudinal physical activity intervention with free-living adults. Overweight adults (*n* = 37; 94.26 ± 17.11 kg; 49.63 ± 12.37 years) were randomly assigned to an intervention (IG, *n* = 17) or usual care group (UC, *n* = 20) as part of a novel nine-month walking intervention. IG participants walked to self-selected music with a predetermined tempo and received a behavioural change support programme. At baseline, four-, six- and nine-months participants were asked to walk around an elliptical track at their habitual pace (0–2 min) and then in time to a predetermined tempo (2–8 min) designed to elicit moderate intensity. Cadence response (steps/min) was assessed and intensity (heart rate (bpm) recorded using wireless telemetry. A repeated measures general linear model (GLM) examined differences between groups over time (*p* < 0.05). All data is presented as means ± SD. At each assessment point both groups displayed an immediate cadence adjustment in response to music tempo (*p* < 0.01) i.e., habitual cadence vs. 3 METs target cadence (*p* < 0.05) and 3 METs target cadence vs. 5 METs target cadence (*p* < 0.05). Additionally, IG participants displayed an increased habitual cadence (0–2 min) at each assessment point (*p* < 0.05; 110 ± 9, 121.80 ± 7.5, 121.46 ± 10, 121.93 ± 7 steps/min respectively). UC participant’s habitual cadence was unchanged from 0–9 months (*p* > 0.05; 120 ± 10, 116 ± 13, 119 ± 12 and 119 ± 9 steps/min respectively). Music tempo may be a useful regulatory tool to prompt the free-living individual to reach an appropriate stride rate to achieve a walking pace that is at least moderate intensity. It also appears that results may be trainable as throughout the study an increased habitual walking cadence was observed, in the absence of music.

## 1. Introduction

Globally, overweight and obesity continue to influence morbidity and mortality rates [1,2]. Physical activity is often prescribed as a method to improve population health by lowering obesity, improving glucose control, and reducing cardiovascular disease and type II diabetes mellitus risk [1,3,4]. Many adult populations find it difficult to introduce and maintain increased physical activity behaviours into their daily routine. Those who strive to attain physical activity recommendations for health are often challenged by adherence issues and accurate interpretation and application of the physical activity guidelines in a real-life setting. Lack of adherence to adequate daily physical activity is often attributed to activities and facilities being inaccessible due to geographical location, too expensive or simply not having the time due to various personal and professional commitments. The misinterpretation regarding physical activity being a form of traditional exercise or sport may also reduce adherence particularly among at risk groups who historically do not enjoy participation in formal exercise or sporting environments. Current physical activity guidelines recommend that physical activity should be at least moderate intensity to elicit health benefits [5,6]. However, it is often difficult for individuals to self-regulate physical activity intensity in a free-living environment [7,8].

Walking is accessible and both cost and time effective. It is also low impact which may be particularly important for individuals who are overweight or obese. Many campaigns have attempted to use walking as a means of increasing physical activity at a population level [9,10]. The 100 step per minute target is commonly promoted as a guideline to help people achieve moderate intensity [11,12,13]. However, achieving moderate intensity may be dependent on many parameters such as cardiorespiratory fitness, motivation, and self-selected free-living pace. Inactive, overweight people may struggle to attain current physical activity recommendations for health, specifically completing at least 30 min of at least moderate intensity activity. A meta-analysis of 41 studies concluded that 143.4 cm/sec (5.16 km/h) was the normal walking pace for men aged 40–44 years [14] which is above the moderate intensity (3 METs) speed when calculated using ACSM metabolic equations [15,16]. The weighted mean results from studies that observed pedestrian cadence under natural conditions (*n* = 8) was 115.2 steps/min [17] which is above the common 100 step/min target [11,13]. However, Tudor-Locke and colleagues explained accelerometer data collected in a large, representative sample suggested that self-selected walking at a cadence equivalent to ≥100 steps/min is a rare occurrence in free-living adults [17,18]. Rowe et al. examined self-selected walking pace of inactive adults and found that when asked to walk ‘briskly’ inactive adults walked at a higher than moderate intensity for bouts of at least 10 min [8]. However, this study was completed under a researcher-controlled environment and as the authors explained, results may have been influenced by the Hawthorne effect [8]. It has been established that cadence or stride rate is influenced by an individual’s height, with taller people taking fewer steps than smaller people over a given distance [17,19]. Therefore, using a global target cadence of 100 steps/min may be inaccurate depending on an individual’s height. It was demonstrated that a height dependent difference of more than 20 steps/min exists in adults achieving moderate intensity (90–113 steps/min for adults 198–152 cm tall, respectively) [19]. To overcome this Rowe et al. developed height-related stride rate guidelines that demonstrate the difference height has on individual stride rate and intensity of activity achieved [19]. It is clear that prompting cadence may be one method of regulating the intensity of walking. Previous research has shown that using a metronome can stimulate cadence response in adults [8,19,20]. However, it is unlikely that this repetitive and tiresome method will be effective over a prolonged period in a free-living environment. Therefore, research must focus on a method of stimulating and regulating walking cadence in a way that is acceptable to the walker, and likely to be effective in the longer term. Novel methods should also aim to increase walking cadence to a level that allows free-living individuals to achieve public health physical activity guidelines i.e., at least moderate intensity. This study aimed to firstly investigate if a predetermined music tempo can prompt participants to achieve a corresponding walking cadence. Secondly, this study aimed to identify if self-selected music with a predetermined tempo, of at least moderate intensity, can provide a stimulus to regulate physical activity intensity in a long-term physical activity intervention with free-living adults.

## 2. Materials and Methods

### 2.1. Participant Characteristics and Randomisation

Ethical approval was granted for this randomised controlled trial from both the Research Ethics Filter Committee, Ulster University and from the Office for Research Ethics Committees Northern Ireland (Approval number: 12/NI/0063). Overweight adults (*n* = 37; 94.26 ± 17.11 kg; 49.63 ± 12.37 years) at risk of developing type II diabetes mellitus were recruited for the current study. Suitable individuals were recruited via NHS Database Reviews (postal invitations), diabetic clinics (clinician led) and from the university population (e-mail and posters). All individuals recruited were screened to assess suitability (recruitment and randomisation details were previously published [21,22]). Interested respondents received a participant information sheet, provided informed consent, and completed a medical history questionnaire before taking part in the research. Participants were randomly assigned to an intervention (IG, *n* = 17) or usual care group (UC, *n* = 20) as part of a nine-month walking intervention [21,22]. IG participants walked to self-selected music with a predetermined tempo based upon Rowe et al. height related stride rate guidelines [19] as detailed below and outlined in Table 1 and received behavioural change support throughout the nine-month study (intervention details were previously published [21]). UC participants received a standard PA information sheet and no additional support.

### 2.2. Development of Walking Programmes and Playlists 

Participants’ moderate intensity cadence was determined using Rowe and colleagues’ height related stride rate cut-points for over ground walking (moderate intensity (3–5 METs) [19]; Table 1). Walking programmes were based on the physical activity guidelines for health. Walking programmes i.e., the frequency, intensity, and duration of each walk, were developed using ACSM guidelines for sedentary adults [16]. Music preferences were recorded e.g., specific artists or genres and music programmes were designed to elicit moderate intensity (based on Rowe et al. guidelines [19]). 

Initially (baseline) music tracks included the top 20 songs from each decade from 1950 to ‘present day’. Each track’s beats per minute was verified in a two-fold manner. Firstly, using beaTunes software (Tagtraum Industries Incorporated, Raleigh, NC, USA) and secondly using a manual tap check. Researchers then manipulated music files by increasing or decreasing the tempo by ± five beats per minute (full details of this process are available on request from the corresponding author). It was noted a change greater than five beats per minute may distort the sound quality. An overall total of 431 tracks were available for selection at baseline. Throughout the study participants selected their preferred music tracks, and/or music genres, or identified other preferences which were made available. 

Playlists were created for each walking bout by using a playlist creator and the Preloaded mobile application. When the app was opened, participants simply viewed their walking programme schedule e.g., walk 1, walk 2 etc. Using this method, the researcher could set each walk duration i.e., length of the playlist, and ensure the correct corresponding target height related stride rate (intensity) i.e., music beat per minute, was predetermined for each individual and in accordance with each participant’s walking programme. Participants were asked to open the Preloaded app, ‘play’ their scheduled walk/playlist and begin their walk simultaneously. Individuals were advised to walk to the beat of the music. Participants were asked to ‘stop’ their playlist once their walk was finished, this ensured completed playlists and associated accelerometer files were saved to the MP3 (iPod touch, Apple Inc., Cupertino, CA, USA) device. Files were assessed using a MATLAB application (The MathWorks Inc., Natick, MA, USA, 2014).

### 2.3. Laboratory Procedures

At baseline, four-, six- and nine-months, participants attended the Ulster University Human Performance Laboratory. Participants’ height (cm), body mass (kg) and body mass index (BMI; kg/m^2^) were assessed on each occasion (methods were previously published [21,22]). Cadence was assessed at each assessment time-point. Participants walked around an elliptical track marked with cones for a total of at least eight minutes. Before beginning the assessment, the protocol was verbally explained to all participants. Participants walked to silence (habitual self-selected tempo) for the first two minutes. Two different music tracks with a predetermined tempo designed to elicit moderate intensity (≥3–5 METs) played via a MP3 player from 2–5 min (track one) and then 5–8 min (track two). Music tempo was predetermined based on the individuals’ height and their corresponding height related stride rate for moderate intensity (3–5 METs) [19]. Cadence response was recorded via the Preloaded app on an MP3 player (iPod touch) and collected data files were later assessed using a MATLAB application. Cadence was also assessed in real-time using hand-counted steps. Walking intensity was recorded using heart rate (bpm) measured using wireless telemetry (Polar Electro, Kempele, Finland).

### 2.4. Statistical Analysis

Levene’s test for equality of variances (*p* > 0.05) were performed to investigate homogeneity of variance in data. Independent-sample *t*-test (*p* < 0.05) were used to compare the means of two groups’ (IG vs. UC) at baseline. Kurtosis, skewness, and Kolmogorov-Smirnov test assessed normality of the distribution. A repeated measures GLM was used to assess the difference between variables with one between-group factor (two groups IG and UC) and two within-group factors (four time points: baseline, four-, six- and nine-months and six music ranges: 0–6 min) (*p* < 0.05). The decision was made to truncate cadence (steps/min) and heart rate (bpm) data to 0–6 min i.e., 0.00 to 6.00 min, as a large percentage (21.1–26.3%) of 6–8-min data i.e., 6.01–8.00 min, was missing. The reason for the missing data ranged from inability to complete the eight minutes of walking, to being injured (21.1–26.3%). Cadence recorded via the Preloaded app was used to confirm real-time hand-counted steps. Spearman’s rank order correlation (rho) was used to examine the relationship between IG and UC stride rate (steps/min) to individual height related stride rate cut points defined previously by Rowe et al. [19]. All data is presented as means ± SD.

## 3. Results

### 3.1. Descriptive Data

A total of 37 participants (*n* = 37; IG *n* = 17; UC *n* = 20) were recruited for the current study. 40.5% of total participants were male and 59.5% female (IG male *n* = 7, female *n* = 10; UC male *n* = 8, female *n* = 12). Following Levene’s test, equality of variances was assumed (*p* > 0.05) for all descriptive data at baseline (age (years, months), height (cm) and body mass (kg)). No significant difference in scores for IG vs. UC participants at baseline were found in any of the following descriptive scores [age (years) t(35) = −0.553, *p* = 0.584; age (months) t(35) = −0.859, *p* = 0.396; height (cm) t(35) = −0.111, *p* = 0.912; body mass (kg) t(35) = 0.486, *p* = 0.630] (Table 2).

Music tracks ranged in tempo from 97 to 113 beats per minute (3 METs) and from 135 to 151 beats per minute (5 METs, Table 3). IG and UC 3 METs mean music tempo was 105.7 ± 4.9 beats per minute and 105.9 ± 4.6. beats per minute, respectively. IG mean music tempo at 5 METs was 143.8 ± 4.9 beats per minute. UC mean 5 METs music tempo was recorded as 143.9 ± 4.6 beats per minute (Table 3).

### 3.2. Cadence (Steps/Min)

**Figure 1:**
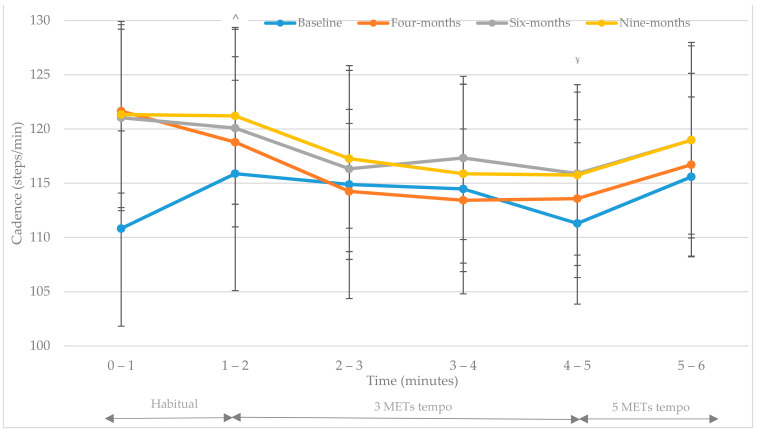
IG (*n* = 17) cadence response to an individualised auditory prompt (music tempo). Repeated measures GLM examined differences between variables (*p* < 0.05). ^ Denotes statistical significance between habitual cadence and 3 METs cadence (*p* < 0.05). ^¥^ Denotes statistical significance between 3 METs cadence and 5 METs cadence (*p* < 0.05).

**Figure 2:**
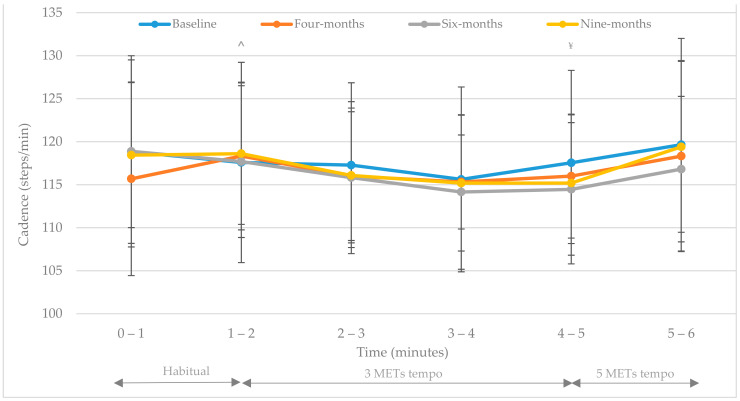
UC (*n* = 20) cadence response to an individualised auditory prompt (music tempo). Repeated measures GLM examined differences between variables (*p* < 0.05). ^ Denotes statistical significance between habitual cadence and 3 METs cadence (*p* < 0.05). **^¥^** Denotes statistical significance between 3 METs cadence and 5 METs cadence (*p* < 0.05).

Levene’s test for equality of variances were assumed (*p* > 0.05) for all cadence (steps/min) data. Means ± SDs for cadence (steps/min; 0–6 min) at each assessment time point are presented in Table 4. A significant interaction (time x group) effect was observed [F(2.7, 94.01) = 3.6, *p* = 0.02, multivariate partial eta squared = 0.09]. There was also a significant difference observed in interaction (time x music x group) effect under different music conditions i.e., habitual cadence (0–2 min), 3 METs target cadence (2–5 min) and 5 METs target cadence (5–6 min) [F(6.7, 233.9) = 2.08, *p* = 0.048, multivariate partial eta squared = 0.56]. Post hoc tests found habitual cadence (0–1 and 1–2 min) was statistically different to 3 METs target cadence (2–3 min, 3–4 min and 4–5 min) (*p* < 0.05). 3 METs target cadence (2–5 min) was also statistically different to 5 METs target cadence (5–6 min, *p* < 0.05). Results were not found to achieve a significant main (time) effect [F(2.69, 233.9) = 2.05, *p* = 0.125, multivariate partial eta squared = 0.54] or a main (group) effect [F(1, 35) = 0.55, *p* = 0.816, multivariate partial eta squared = 0.56]. The training effect observed in the results above is displayed in Figure 1 and Figure 2. 

At each assessment point both groups displayed an immediate cadence adjustment in response to music tempo (*p* < 0.01). This acute response was greater and more prolonged in IG participants during each assessment (Figure 1 and Figure 2). Figure 1 and Figure 2 illustrate participants’ habitual (unprompted) cadence from baseline to nine-months (Table 4). A significant difference was observed between groups habitual cadence at baseline (t(35) = 2.45, *p* = 0.02; Table 4). Although non-significant between groups at four-, six- and nine-months (*p* > 0.05; Table 4) IG participants displayed a significantly increased habitual cadence (0–2 min i.e., without music) between baseline and four-months (*p* = 0.003), baseline and six-months (*p* = 0.015) and baseline and nine-months (*p* = 0.022; 110.8 ± 9.0 steps/min, 121.6 ± 7.6 steps/min, 121.0 ± 8.6 steps/min, 121.3 ± 8.6 steps/min respectively; Figure 1 and Table 4). UC participant’s habitual cadence displayed no significant change from baseline throughout the study (118.8 ± 10.7 steps/min, 115.7 ± 11.3 steps/min, 118.9 ± 11.1 steps/min, 118.4 ± 8.4 steps/min at baseline, four-, six- and nine-months respectively: Figure 2 and Table 4). 

### 3.3. Heart Rate (bpm)

Levene’s test for equality of variances were assumed (*p* > 0.05) for all heart rate (bpm) data. No significant interaction (time x group) effect [F(2.7, 94.3) = 2.21, *p* = 0.098, multivariate partial eta squared = 0.59] and no significant main effect (time) [F(2.7, 94.3) = 0.881, *p* = 0.444, multivariate partial eta squared = 0.25] was observed for heart rate response. A significant interaction (time × music) effect was observed [F(5.7, 200.5) = 3.2, *p* = 0.006, multivariate partial eta squared = 0.84] but not in (time x music x group) [F(5.7, 200.5) = 1.95, *p* = 0.078, multivariate partial eta squared = 0.53]. No significant main (group) effect was observed [F(1) = 0.146, *p* = 0.705, multivariate partial eta squared = 0.004]. Post hoc tests identified heart rate (bpm) response was significantly different (*p* < 0.001) under all of the target cadences (music conditions) i.e., heart rate (bpm) response during habitual cadence (0–2 min), during 3 METs target cadence (2–5 min) and during 5 METs target cadence (5–6 min). Heart rate (bpm) data are presented in Table 4. 

### 3.4. Cadence (Steps/Min) vs. Predetermined Height Related Stride Rate Cut-Points (3 METs and 5 METs Target)

**Figure 3:**
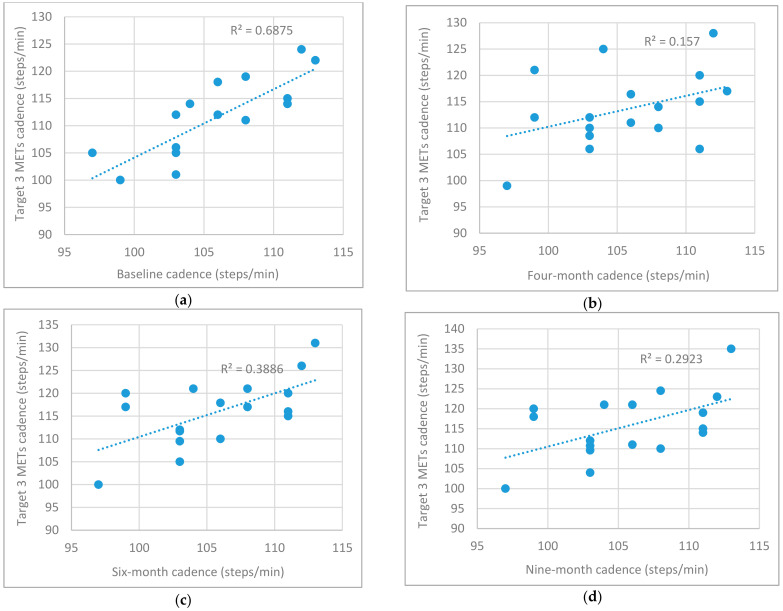
(**a**–**d**) Target cadence (3 METs; steps/min) and actual cadence (steps/min) response at each time point for IG (*n* = 17) participants.

**Figure 4:**
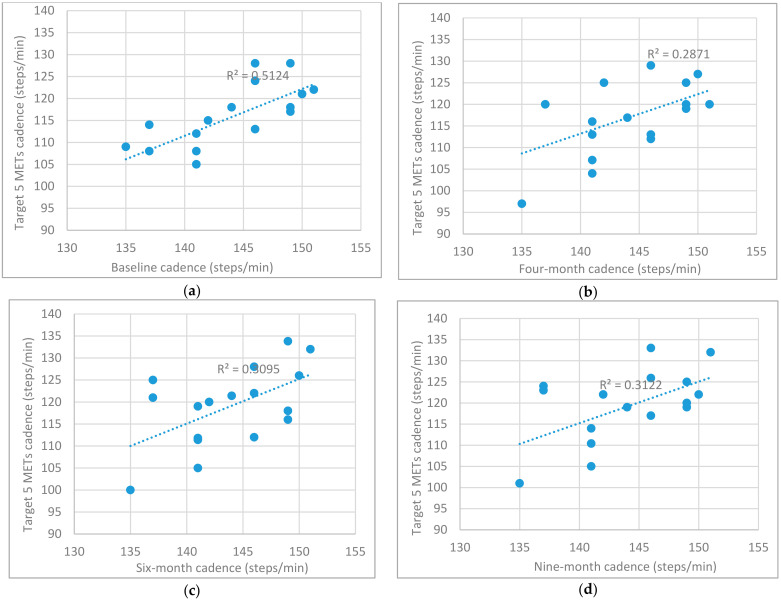
(**a**–**d**) Target cadence (5 METs; steps/min) and actual cadence (steps/min) response at each time point for IG (*n* = 17) participants.

Spearman’s rank order correlation was used to examine the relationship between IG and UC cadence (steps/min) and individual predetermined height related stride rate cut points defined previously by Rowe et al. [19]. There was a strong, positive relationship found between 3 METs and 5 METs target cadence (steps/min) and actual cadence (steps/min) completed by IG participants at baseline, four-months (5 METs target only), six-months and nine-months (*p* < 0.05; actual cadence vs. 3 METs target: Baseline [r = 0.86, *n* = 17, *p* < 0.001]; six-months [r = 0.59, *n* = 17, *p* = 0.013]; nine-months [r = 0.54, *n* = 17, *p* = 0.026]; actual cadence vs. 5 METs target: Baseline [r = 0.75, *n* = 17, *p* < 0.001]; four-months [r = 0.50, *n* = 17, *p* = 0.043]; six-months [r = 0.53, *n* = 17, *p* = 0.028]; nine-months [r = 0.50, *n* = 17, *p* = 0.043]; Table 5). A moderate positive correlation was found between IG four-month cadence and 3 METs target cadence [r = 0.40, *n* = 17, *p* = 0.114]. UC participants recorded small correlations to both 3 METs and 5 METs cadence targets which varied in direction i.e., ‘+’ and ‘−’ and none of which were statistically significant (*p* > 0.05; Table 5, Figure 3 and Figure 4). 

## 4. Discussion

The main finding of this study was that predetermined music tempo can prompt participants to achieve a corresponding walking cadence. Secondly, this study found that music with a predetermined tempo may be a useful tool to provide a stimulus to regulate walking intensity in free-living adults. These effects appear to be trainable and are sustained in the absence of an auditory prompt (music), once trained.

Previous research has shown using a metronome as an auditory prompt can help individuals to achieve a predetermined walking cadence [19,20]. However, this monotonous stimulus may become tiresome over time. More recently, Perry et al. modulated the tempo of a single commercial song to entrain light (<3 METs, 80 beats per minute), moderate (3.0 to 5.9 METs, 100 beats per minute) and vigorous (≥6.0 METs, 125 beats per minute) intensity walking [23]. The authors concluded that music entrainment overall increased metabolic intensity at a given cadence compared with the self-paced walking trials in healthy young adults [23]. In contrast to Perry and colleagues who examined six five-minute walking conditions (with and without music) on one test occasion [23], this study examined the effects of walking to music with an individualised beat, based on Rowe et al. height related stride rate guidelines [19], across nine months. The current study found that by using self-selected music with a predetermined tempo of at least 3 METs in accordance with Rowe et al. height related stride rate cut-points [19] participants’ cadence was successfully manipulated. 

The findings demonstrated the immediate cadence response to music tempo (3 METs and 5 METs targets) (Figure 1 and Figure 2) provided an auditory stimulus which significantly regulated walking cadence of overweight adults (habitual cadence vs. 3 METs target cadence, *p* < 0.05; 3 METs vs. 5 METs target cadence, *p* < 0.05). At each assessment point both groups displayed an immediate cadence adjustment in response to music tempo (*p* < 0.05). This acute response was greater in IG participants during each assessment (Figure 1 and Figure 2 and Table 4). IG cadence response to music differed greatly at four-, six- and nine-months in comparison to baseline cadence results recorded at each walking intensity (habitual, 3 METs and 5 METs target cadence; Figure 1). In contrast UC cadence response was similar across all time points (Figure 2). Styns et al. investigated the immediate effects of walking while listening to music in comparison to metronome stimuli [20]. These authors found participants walked faster when listening to music in comparison to metronome stimuli [20]. They also observed an increased walking speed as the auditory tempo increased, regardless of participants’ ability to synchronise movements. Styns and colleagues reported most participants (72.6%) regulated their cadence within one beat of the stimulus (music or metronome) [20]. Similarly, Perry et al. found in 90% of participants entraining to a music tempo of 100 beats per minute yielded ≥3 METs i.e., at least moderate intensity [23]. In addition, a recent publication from the current research group found 71.4%, 79.5% and 73.3% of IG walking completed during the free-living nine-month study was at least moderate intensity i.e., ≥predetermined target cadence, in a free-living environment between baseline to four-months, four- to six-months, and six- to nine-months, respectively [22]. One of the most interesting findings of the current study highlighted that walking to self-selected music with a predetermined tempo [19] influenced IG participants’ habitual cadence i.e., cadence intensity was maintained in the absence of the auditory prompt (Figure 1). UC participant’s habitual cadence displayed no significant change from baseline to nine-months (baseline 118.84 ± 10.67 steps/min; four-months 115.68 ± 11.25 steps/min; six-months 118.88 ± 11.11 steps/min; nine-months 118.44 ± 8.43 steps/min). Therefore, cadence response appears trainable as displayed in the significant difference observed within IG walking baseline values vs. other assessment points (Table 4). In contrast UC participants’ walking cadence response displays little change with similar patterns observed at each assessment (Figure 2). In particular, the increase of habitual (unprompted) cadence from baseline to all other assessment points (four-, six- and nine-months) in IG participants, as discussed previously, suggests a training effect did take place (*p* < 0.05). Future research should be completed to confirm these results in a larger cohort however it may be suggested these findings might have implications for free-living individuals. Firstly, these findings suggest walking to music with a predetermined individualised tempo can help adults to regulate their cadence. This would have implications in a free-living environment as walking to music with a predetermined tempo may provide a method to attain the desired walking intensity to meet current physical activity guidelines. Secondly, these findings appear to demonstrate a training effect which would be beneficial in a free-living environment to regulate exercise intensity. These results not only suggest music can train individuals’ cadence to achieve a predetermined tempo, but also determine that cadence enhancements achieved remain in the absence of the auditory prompt. This finding is significant as it provides a realistic and accessible tool for free-living individuals to ensure walking is at the recommended intensity to achieve physical activity guidelines for health [5,6]. However, as participants’ habitual cadence was assessed using their self-selected or preferred cadence within a controlled research setting there is no way of knowing from the current results whether this reflects their actual habitual cadence in a free-living environment. In addition, the potential influence of the Hawthorne effect must be acknowledged as participants habitual cadence observed is greater than that found in previous research e.g., 100 steps/minute [18]. Therefore, care should be taken when interpreting these results and future research should aim to replicate this study within a free-living environment and assess habitual cadence without the influence of researcher observation.

Interestingly, IG participants’ habitual (unprompted) cadence (steps/min) at four-, six- and nine-months is greater than that determined for 3 METs and 5 METs target cadences (Figure 1). These results show that a training effect occurred overtime but also that the habitual cadence, following periods of walking to music with predetermined tempos i.e., during the intervention, is not only higher than baseline habitual (unprompted) values but also greater than auditory prompted cadence responses (3 METs and 5 METs targets) at each assessment (four-, six- and nine-months). This may suggest although music prompts adults to achieve a predetermined cadence and regulates walking to at least moderate intensity (3 to 5 METs) it may overtime lead to a higher habitual cadence than the predetermined target tempo. However, this may not be an issue as IG habitual cadence observed in the current study was greater than the minimum 3 METs moderate intensity threshold which physical activity recommendations promote i.e., at least moderate intensity [5,6]. The clinical benefits and health adaptations of maintaining a greater habitual cadence during non-auditory prompted physical activity requires further research however it is safe to postulate that it is likely to provide greater health benefits than walking at a lower cadence and therefore a lower exercise intensity. Additionally, whether this observed greater habitual cadence is a result of participants simply walking faster as they adapt to physical activity and improve their cardiovascular fitness or as discussed earlier is a consequence of the research environment requires more research. It is however clear that music tempo may be used as a useful tool to prompt adults to achieve a corresponding walking cadence and provide a stimulus to regulate walking intensity in a free-living environment which is at least moderate intensity. Previous research [18] found ambulatory overweight and obese U.S adults’ peak one minute cadence ranged from 87.6 to 102.2 steps/min which is lower than that displayed by both IG and UC participants’ habitual cadence (Table 4). These authors concluded that peak one minute cadence displayed significant and consistent declines with age and increasing levels of obesity [18]. The lower peak cadence results found in this U.S cross-sectional study [18] in contrast to the current study’s habitual cadence findings may be attributed to methodological differences i.e., although the data was collected over one year and from a much larger sample size (*n* = 3522) it did not form part of a randomised control trial i.e., there was no intervention. More recently Tudor-Locke and colleagues reinforced the global 100 steps/min public health message for use in adult populations [11]. However, using the methods of the current study i.e., self-selected music with an individualised tempo, may provide a simple solution to individualising population-based physical activity guidelines which would enable individuals to regulate physical activity to at least moderate intensity in a real-world setting.

This cadence training argument is strengthened based on the correlation results which found IG participants actual cadence (steps/min) displayed a strong, positive relationship to both 3 METs and 5 METs target cadences at all intervention assessment-points. The only exception was recorded for IG four-month cadence (steps/min) vs. 3 METs target. However, a moderate, positive correlation was still found between these variables [r = 0.40]. In contrast all UC cadence (steps/min) for both targets (3 METs and 5 METs) displayed small correlations of varying directions (positive and negative), none of which recorded statistical significance (*p* > 0.05). These results compare favourably to Tudor-Locke and colleagues review of controlled studies of ambulatory activity using treadmills, tracks, and/or hallways which also found a strong, positive correlation between cadence and intensity (METs) [r = 0.94] [24]. These results demonstrate music tempo firstly prompted IG participants to achieve the desired target cadence and secondly allowed regulation of exercise intensity (3 METs and 5 METs) within this group. It is clear from these results that IG participants accurately regulated their cadence in response to the predetermined music tempo in contrast to the UC which displayed no correlation to the predetermined tempos (3 METs or 5 METs).

Moderate intensity is widely accepted as activities ≥3 METs [15,16]. It should be noted that use of these metabolic equations to estimate energy expenditure have their limitations for example they have been found to underestimate walking energy expenditure and do not account for individual differences such as cardiorespiratory fitness or age [8,25,26,27]. Walking intensity in the current study was at least moderate (≥3 METs) among IG participants and intensity was regulated throughout the study as discussed previously. Tudor-Locke and colleagues suggested although public health physical activity guidelines have traditionally focused on promoting a detailed exercise prescription, adults should aim to attain ≥7500 steps/day, of which ≥3000 steps (representing at least 30 min) should be completed at a cadence ≥100 steps/min [11,13]. All participants in the current study achieved a cadence ≥100 steps/min during each walking stage at each assessment point (baseline, four-, six- and nine-months) suggesting this population are capable of attaining physical activity recommendations for health [5,6]. However, as noted this data was recorded in a controlled environment. Walking and intensity compliance (%) of this novel nine-month randomised controlled trial have been previously reported [21,22]. Walking compliance from baseline to four-months was 70.1% ± 39.17% (range 9.0% to 158.4%). This was followed by decreases to 43.4% ± 56.1% (range 0% to 225%) between four- to six-months and to 37.5% ± 43.5% (range 0% to 125.3%) at nine-months which as explained in previous publications, may be reflective of the reduced behaviour change support at these timepoints [21,22]. Despite the decreases reported in walking compliance, % intensity compliance i.e., achieving the predetermined target cadence of at least moderate intensity, of the walks completed was ≥71.4% throughout the study. This suggests attaining moderate intensity activity within this population is achievable. Therefore, future research should focus on the ability of this population to achieve ≥100 steps/min for at least 150 min per week in a free-living environment. The effects of sustained behaviour change support on walking compliance should also be further explored.

Current physical activity guidelines for health recommend adults achieve at least 150 min of moderate to vigorous physical activity per week [5,6]. However, some challenges exist for individuals attempting to achieve these recommendations. Firstly, many adults simply do not meet the recommended prescription i.e., do not complete the required physical activity frequency, intensity, time, or volume. Secondly, when individuals appear to meet the guidelines e.g., achieve ≥ at least 150 min of moderate intensity activity per week, often the intensity of physical activity performed is below the moderate to vigorous recommendation. As discussed, the U.S. National Health and Nutrition Examination Survey (NHANES) results suggested that self-selected walking at a cadence equivalent to ≥100 steps/min is a rare occurrence in free-living adults [18]. However, in a recent systematic review of 14,015 participants, Murtagh and colleagues reported a usual outdoor walking cadence of 116.65 steps/min in ambulatory, apparently healthy, and community-dwelling adults (>18 years) [28]. Therefore, free-living populations require a reliable objective tool to help them achieve at least moderate intensity in their daily routines. Finally, although promotion of the current population-based guidelines is undoubtedly significantly important from a clinical perspective, they are a global recommendation and not individualised. This study demonstrated that employing music to regulate walking intensity is successful in a free-living adult population. Moreover, if the walker attains at least the predetermined individualised tempo then it can be said that music may be used in a free-living environment to achieve at least moderate intensity. Furthermore, as the music tempo is tailored based on height then the intensity is also individualised, which moves from the current global ‘one size fits’ all physical activity guidelines. 

Heart rate (bpm) increased concomitantly as the target intensity of each walking stage increased (3 METs and 5 METs). This would be expected as it reflects a greater cardiac strain associated with each increase in physical activity intensity. Although similar heart rate (bpm) responses were recorded for both groups at baseline and no significant difference was observed between groups, IG heart rate (bpm) response displayed a lowering trend in contrast to UC participants across other intervention assessment points (Table 4). Therefore, it may be suggested for a given walking intensity UC participants were exerting more cardiovascular strain. IG heart rate (bpm) response achieved in each walking stage increased non-significantly from baseline to the end of the six-month intervention. However, this increase was accompanied by an increased cadence response (Table 4), suggesting improved cardiovascular ability and adaptation to exercise. It may be postulated that an improved cardiovascular fitness may be due to increased stroke volume which results in a greater volume of blood being pumped from the heart with each beat. This in turn facilitates activity longevity and prevents fatigue as more oxygen is carried to the working muscles and enhances removal of by-products which influence fatigue. In contrast UC heart rate (bpm) response was sporadic without significant changes in cadence suggesting no cardiovascular fitness gains. Hills et al. [29] reported obese participants when asked to ‘walk for pleasure’ exerted a greater cardiovascular strain at these self-selected walking intensities in contrast to their non-obese counterparts although both groups perceived rate of exertion was similar [29]. These authors reported when asked to ‘walk for pleasure’ moderate intensity walking was performed, although obese participants engaged in lower scale moderate intensity walking [29]. This reflects the findings of the current study which demonstrated walking of at least moderate intensity is sufficient to stimulate cardiovascular stress via increased heart rate response in overweight adults which may promote beneficial health adaptations. 

While this study has novel qualities it is not without its limitations. To the authors knowledge this is the first study to investigate if walking to self-selected music with a predetermined tempo is an effective way to enable free-living adults to achieve a desired cadence. However, music has many characteristics e.g., pulse clarity and volume which may have an effect on findings. Future research should aim to explore the effects of other characteristics of music on similar populations and study design. This study also identified that walking to music enables free-living adults to regulate cadence and undertake physical activity of at least moderate intensity. However, another limitation was that the cardiorespiratory response to walking at a predetermined tempo was not analysed in the current study. Although the effects of walking to METs determined intensity were examined, as discussed there are limitations associated with this method of intensity identification. While it is acknowledged due to resources (physical and time) researchers often work within such constraints, future research should examine the use of indirect calorimetry to identify exact energy expenditure requirements for predetermined target tempos. The need for habitual cadence to be assessed in a free-living environment also warrants further investigation. Furthermore, the impact of behaviour change support throughout a longitudinal study on compliance merits further study within this population. Aside from the rhythmic regulation of walking cadence outlined in the current study, music may also provide additional benefits to the walker. Physical benefits may include efficiency of gait, with improvements in gait walking becomes more fluid and may allow individuals to complete a given distance in a quicker time i.e., increase speed [30]. Karageorghis and Priest found music encouraged the movement pattern to synchronise with the beat of the music and influenced psychomotor arousal [31,32]. Parallel to physical effects music has been shown to have motivational effects during exercise and walking [33,34,35] and may decrease one’s rate of perceived exertion [31,32] allowing them to attain a higher intensity output and or over a prolonged period. Music has also been found to have a positive effect on mood [31,32] and when used during walking improved mood may have a significant effect on adherence to exercise particularly within at-risk populations such as obese adults [35]. Terry et al. found music provided ergogenic, psychological, and physiological benefits in a laboratory-based study on elite triathletes [34]. These authors concluded the motivational qualities of music may be less important than the prominence of its beat and the degree to which participants are able to synchronise their movements to its tempo [34]. Although these researchers applied the use of music to an elite group of athletes the same may be suggested for at risk populations trying to attain physical activity recommendations for health benefits. Future research should investigate the long-term benefits of using music as a method to regulate tempo and perhaps improve physical activity adherence within overweight and obese populations. 

## 5. Conclusions

Prescribing walking as a mode of physical activity is a method to achieve physical activity guidelines and improve health within overweight adult populations. As walking is a non-discriminant activity, accessible and free it is likely to improve adherence rates within this population. Music tempo may be a useful regulatory tool to prompt free-living individuals to achieve an appropriate stride rate to attain a walking pace that is at least moderate intensity. Furthermore, using Rowe et al. height relate stride rate cut-points [19] is a method to ensure that the intensity remains individualised based on height. Using self-selected music with a predetermined tempo individualises walking intensity and ensures populations are striving to achieve an individual based cadence of at least moderate intensity instead of a universal target e.g., ≥100 steps per minute. The current study’s results highlight that a cadence training effect occurs and in the absence of music as an auditory cue, habitual cadence remains at an intensity ≥3 METs.

Currently adult populations struggle to meet physical activity guidelines for health [4,5,36]. When adults strive to adopt these guidelines, they particularly struggle to regulate intensity in a free-living environment [24]. The findings of the present study help to address these current issues with physical activity public health guidelines in a two-fold manner. Firstly, walking to music may help to improve adherence to physical activity through achieving additional benefits such as increased enjoyment, motivation and decreased perceived exertion for a given intensity. Secondly, walking to music may help free-living individuals to regulate their physical activity intensity in the free-living environment. Future research should aim to replicate this methodology in longitudinal studies with similar populations.

## Figures and Tables

**Table 1:** Stride rate (steps per minute) cut points for adults of different heights, corresponding to 3, 4, and 5 METs. Adapted from Rowe et al. [19].

Height (Inches)	Height (cm)	Stride Rate (3 METs)	Stride Rate (4 METs)	Stride Rate (5 METs)
Generic ^a^		103	122	141
60	152.4	113	132	151
61	154.9	112	131	150
62	157.5	111	130	149
63	160.0	109	128	147
64	162.6	108	127	146
65	165.1	107	126	145
66	167.6	106	125	144
67	170.2	104	123	142
68	172.7	103	122	141
69	175.3	102	121	140
70	177.8	100	119	138
71	180.3	99	118	137
72	182.9	98	117	136
73	185.4	97	116	135
74	188.0	95	114	133
75	190.5	94	113	132
76	193.0	93	112	131
77	195.6	91	110	129
78	198.1	90	109	128

^a^ General recommendations based on all heights.

**Table 2:** Descriptive variables between groups at baseline.

Variable	Group	(*n*)	Mean	SD
Age (years)	IG	17	48.53	9.87
	UC	20	50.75	13.81
Height (cm)	IG	17	168.18	10.10
	UC	20	168.53	9.20
Body mass (kg)	IG	17	96.24	15.66
	UC	20	93.48	18.45

**Table 3:** Summary of music tempo (beats per minute) at 3 METs and 5 METs intensities.

Group (*n*)	Music Tempo (Beats Per Minute) 3 METs				Music Tempo (Beats Per Minute) 5 METs			
	Mean	SD	Range (Min)	Range (Max)	Mean	SD	Range (Min)	Range (Max)
IG (*n* = 17)	105.7	4.9	97	113	143.8	4.9	135	151
UC (*n* = 20)	105.9	4.6	99	112	143.9	4.6	137	150
Overall (*n* = 37)	105.8	4.7	97	113	143.8	4.7	135	151

**Table 4:** Cadence (steps/min) and heart rate (bpm) response in IG and UC participants.

Assessment Time-Point	Cadence Target	Time (Mins)	Cadence (Steps/Min)						Heart Rate Response (bpm)					
			IG (*n* = 17)		UC (*n* = 20)		Overall (*n* = 37)		IG (*n* = 17)		UC (*n* = 20)		Overall (*n* = 37) ^$^	
			Mean	SD	Mean	SD	Mean	SD	Mean	SD	Mean	SD	Mean	SD
Baseline	Habitual	0–1	110.82 ^†^	9.00	118.85 ^†^	10.67	115.16	10.61 ^^^	106	13	108	13	107	13
		1–2	115.88	10.78	117.70	11.64	116.87	11.14 ^^^	112	15	112	15	112	15
	3 METs	2–3	114.88	10.52	117.26	9.58	116.17	9.95 ^^,¥^	116	18	114	14	115	15
		3–4	114.47	9.67	115.73	10.74	115.15	10.14 ^^,¥^	118	17	116	14	117	15
		4–5	111.29	7.44	117.67	10.74	114.74	9.79 ^^,¥^	120	18	117	14	119	16
	5 METs	5–6	115.59	7.37	119.81	12.36	117.87	10.45 ^¥^	122	17	119	17	121	17
Four-months	Habitual	0–1	121.64 ^ǐ^	7.56	115.95	11.25	118.57	10.02 ^^^	107	10	112	7	110	9
		1–2	118.78	5.71	118.56	8.58	118.66	7.31 ^^^	111	11	116	9	114	10
	3 METs	2–3	114.25	6.27	116.26	7.48	115.34	6.93 ^^,¥^	111	13	118	10	115	12
		3–4	113.43	6.58	115.70	5.47	114.66	6.03 ^^,¥^	112	14	120	10	116	12
		4–5	113.58	7.28	116.34	7.21	115.08	7.27 ^^,¥^	114	15	120	10	117	12
	5 METs	5–6	116.71	8.43	118.71	11.10	117.79	9.88 ^¥^	116	16	122	11	119	14
Six-months	Habitual	0–1	121.04 ^ǐ^	8.57	119.33	11.12	120.12	9.93 ^^^	108	15	109	11	109	13
		1–2	120.08	9.11	118.25	8.83	119.09	8.88 ^^^	114	16	114	14	114	15
	3 METs	2–3	116.33	5.47	116.08	8.83	116.20	7.38 ^^,¥^	117	20	118	12	118	16
		3–4	117.33	7.52	114.44	8.98	115.77	8.36 ^^,¥^	118	21	120	15	119	18
		4–5	115.88	7.52	114.84	8.66	115.32	8.06 ^^,¥^	118	20	121	14	119	17
	5 METs	5–6	118.96	9.01	117.27	8.46	118.05	8.64 ^¥^	121	22	123	15	122	18
Nine-months	Habitual	0–1	121.33 ^ǐ^	8.59	118.82	8.43	119.98	8.48 ^^^	108	12	108	10	108	11
		1–2	121.21	8.15	118.98	8.23	120.01	8.16 ^^^	112	14	112	11	112	12
	3 METs	2–3	117.27	8.58	116.37	7.84	116.78	8.08 ^^,¥^	115	17	114	12	114	14
		3–4	115.88	8.24	115.53	7.90	115.69	7.95 ^^,¥^	116	16	115	12	115	14
		4–5	115.75	8.33	115.53	7.03	115.63	7.54 ^^,¥^	117	15	116	12	117	13
	5 METs	5–6	118.98	8.68	119.64	9.93	119.34	9.26 ^¥^	119	18	120	15	119	16

Repeated measures GLM examined differences between variables (*p* < 0.05). ^†^ = denotes statistical difference between groups for given variable (*p* < 0.05); ǐ = denotes a within group statistical significance between given value and baseline value (*p* < 0.05); ^^^ = denotes significant difference between habitual cadence and 3 METs target cadence scores (*p* < 0.05); ^¥^ = denotes significant difference between 3 METs and 5 METs target cadence scores (*p* < 0.05); ^$^ = denotes significant interaction effect (time x music) between heart rate (bpm) response across all target cadences i.e., heart rate response to habitual vs. 3 METs target vs. 5 METs target cadences (*p* < 0.05).

**Table 5:** Correlations between target cadence and actual cadence (steps/min).

Assessment Time-Points (Actual Cadence, Steps/Min)	IG (*n* = 17)	UC (*n* = 20)	IG (*n* = 17)	UC (*n* = 20)
	3 METs Target Cadence (Steps/Min)	3 METs Target Cadence (Steps/Min)	5 METs Target Cadence (Steps/Min)	5 METs Target Cadence (Steps/Min)
Baseline (steps/min)	0.86 **	0.03	0.75 **	0.12
Four months (steps/min)	0.40	0.03	0.50 *	0.19
Six months (steps/min)	0.59 *	0.11	0.53 *	0.30
Nine months (steps/min)	0.54 *	−0.09	0.50 *	0.18

** = Correlation is significant *p* < 0.001; * = Correlation is significant *p* < 0.05.

## Data Availability

Data may be made available upon request.
